# 4241 Identification of small molecules that facilitate the efficient differentiation of stem cell derived β-cells

**DOI:** 10.1017/cts.2020.69

**Published:** 2020-07-29

**Authors:** Yuhao Min, Chris Clifford, Quinn P. Peterson

**Affiliations:** 1 Mayo Clinic

## Abstract

OBJECTIVES/GOALS: In this study, we established a high-throughput chemical screening platform to identify small molecules that facilitates efficient differentiation of stem cells derived β (SC-β) cells. Using this platform, we identified several compounds that potentially increase the differentiation efficiency. METHODS/STUDY POPULATION: Differentiation of human embryonic stem cells (HUES8) into SC-β was carried out using previously published protocols in a 3D cell suspension. Single cells were replated in Matrigel-coated well plates at the start of different stages depending on experiments. Differentiation medium supplemented with small molecules at a final concentration of 2 M and 0.2 M was used throughout the stage. All the cells were then fixed and permeabilized. Immunocytochemical staining was performed. Images of each well were taken and analyzed. Numbers of the total cell, insulin-positive cell, NKX6.1-positive cell, and co-positive cell were recorded. Candidate compounds were validated using flow cytometry or ICC. RESULTS/ANTICIPATED RESULTS: We identified several hit compounds that significantly increase the NKX6.1 positive cell percentage compared to the DMSO-treated controls when treated at the PP1 cell stage. Follow up assays demonstrated that at least one of these putative hits reproducibly increased NKX6.1 expression. In addition, we identified other compounds that significantly increase the insulin and NKX6.1 copositive SC-β cell population when treated at the later PP2 cell stage during the differentiation. We expect a dosage-dependent response when the candidate hits are validated using more accurate assays. DISCUSSION/SIGNIFICANCE OF IMPACT: We established a high-throughput screening platform to identify small molecules that increase the efficiency of SC-β direct differentiation. Successful generation of SC-β allows cell replacement therapy in diabetes patients, and a better understanding of pancreatic biology and development.

